# Stricter correction of leg length discrepancy is required during total hip arthroplasty in patients with ankylosing spondylitis

**DOI:** 10.1186/s12891-023-06908-7

**Published:** 2023-10-03

**Authors:** Chae-Jin Im, Chan Young Lee, Jae Young Beom, Min-Gwang Kim, Taek-Rim Yoon, Kyung-Soon Park

**Affiliations:** Department of Orthopaedic Surgery, Chonnam National University Medical School and Hospital, Seoyang-ro 322, Hwasun-gun, Chonnam, 58128 Republic of Korea

**Keywords:** Ankylosing spondylitis, Coronal balance, Leg length discrepancy, Total hip arthroplasty

## Abstract

**Background:**

Patients with ankylosing spondylitis often have fusions in the spine and sacroiliac joints, such that it is difficult to compensate for leg length discrepancy (LLD).

**Methods:**

We retrospectively measured the LLD after total hip arthroplasty (THA) in 89 patients with ankylosing spondylitis from June 2004 to February 2021 at our institute. Patients were divided into two groups based on an LLD of 5 mm. Clinical outcomes were investigated using the Western Ontario and McMaster Universities Osteoarthritis Index (WOMAC) and Harris Hip Score (HHS). In addition, these points are investigated: patient satisfaction with the operation; whether there was a current difference in leg length; and whether there was a limping gait.

**Results:**

The group with an LLD of 5–10 mm rather than < 5 mm had significantly worse WOMAC pain and stiffness. The survey revealed statistically significant differences in patient satisfaction with the operation, limping gait, and whether back pain had improved.

**Conclusion:**

For patients with ankylosing spondylitis, reducing the LLD to < 5 mm, which is more accurate than the current standard of < 10 mm, may produce greater improvement in clinical outcomes after hip arthroplasty.

## Introduction

Ankylosing spondylitis (AS) is a chronic inflammatory disease that causes inflammation in multiple joints, more frequently in the spine and sacroiliac joints. Spinal fusion often occurs in patients with advanced AS, and patients with AS have reduced flexibility in their vertebral joints [1]. Inflammation in the hip joint occurs in about 24–36% of cases, and total hip arthroplasty (THA) is the preferred surgical treatment for moderate-to-severe hip arthritis [2]. AS has a higher incidence of various complications after THA than in patients undergoing THA for other diseases [3]. A leg length discrepancy(LLD) is recognized in about 30% of patients after THA [4]. The degree of discomfort has been reported to vary with differences of 5–10 mm in lower extremity length after THA [5]. Many patients with AS experience discomfort due to an imbalance of the lower extremities during walking, or an imbalance of the whole body when standing, after THA. There has been no study on the extent to which the LLD due to THA causes discomfort in patients with AS. The purpose of this study was to compare the difference in functional indicators after surgery in patients with ankylosing spondylitis by dividing the patients into groups based on leg length discrepancies (LLDs) observed at follow-up after THA.

## Materials and methods

This study was approved by the Institutional Review Board of Chonnam National University Hwasun Hospital. All procedures of this study were carried out in accordance with relevant guidelines and regulation. The need for informed consent was waived by the Institutional Review Board of Chonnam National University Hwasun Hospital, because of the retrospective nature of the study. Patients’ clinical and radiographic data was collected through the hospital’s medical information system.

### Patients

The inclusion and exclusion criteria for this retrospective study were as follows:

Inclusion criteria:

1. Patients diagnosed with ankylosing spondylitis who underwent THA at Chonnam National University Hwasun Hospital from June 2004 to February 2021.

Exclusion criteria:

1. Patients for whom the LLD could not be determined on X-ray (due to fused hip).
2. Patients who did not have a follow-up imaging examination after ≥ 6 weeks post-operation.
3. Patients who underwent revision surgery due to implant failure, which could potentially influence clinical outcome.
4. Non-responders to the survey.

**Fig. 1:**
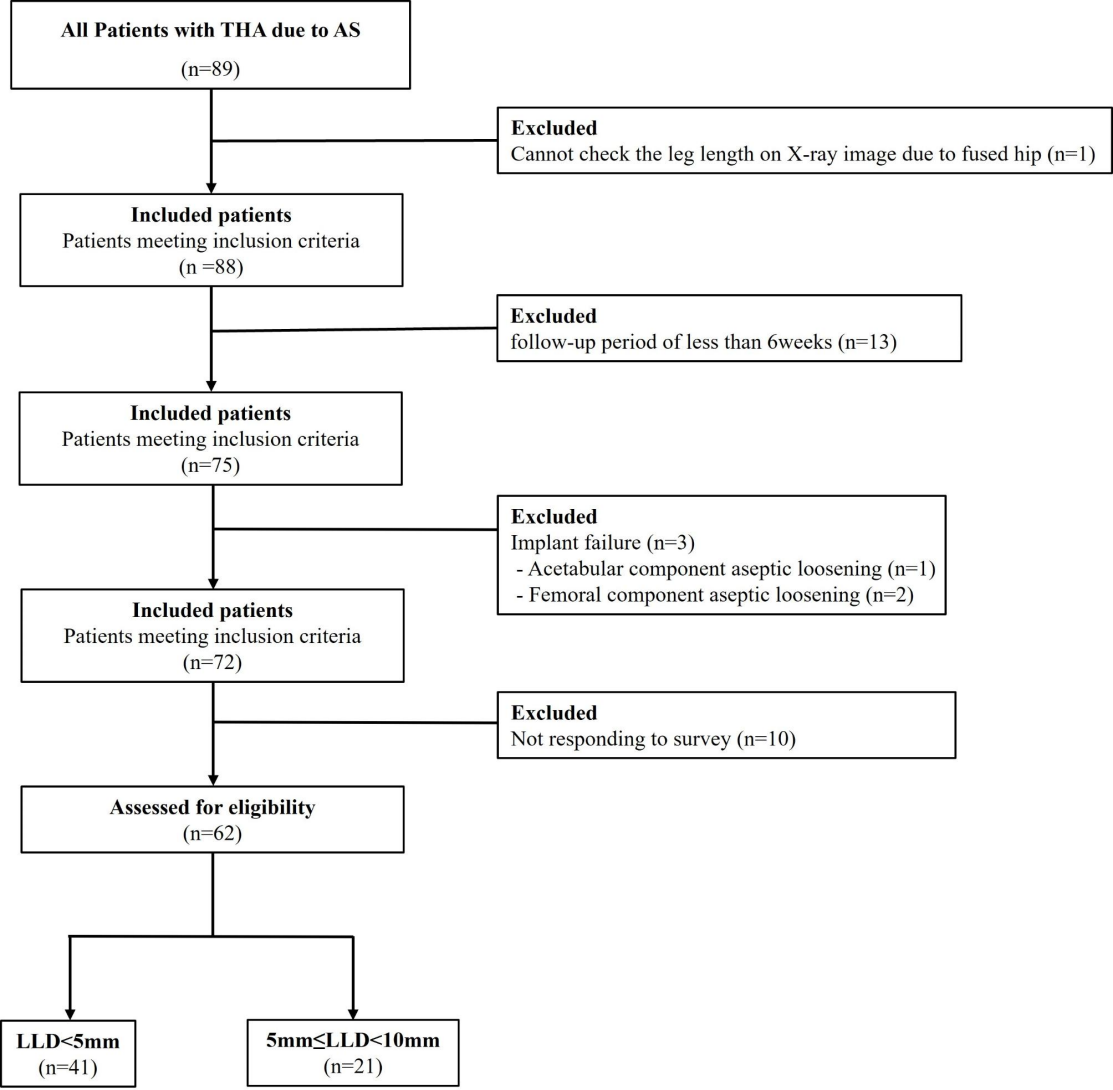
Flowchart describing the patient enrollment process. THA = total hip arthroplasty; AS = ankylosing spondylitis; LLD = leg length discrepancy

After applying the inclusion and exclusion criteria, a total of 62 patients were enrolled in the study. A flowchart detailing the selection process can be found in Fig. 1.

### Surgical techniques

All patients were operated on by two high-volume arthroplasty surgeons using one of two approaches. The modified minimally invasive two-incision approach was used in 51 cases (82%), and the posterolateral approach was used in 11 cases (18%) in the lateral decubitus position(p = 0.110) [6, 7]. For patients with large LLDs or a prior surgical history, the posterolateral approach was used. Cementless femoral and acetabular component with ceramic-on-ceramic bearing were used in all patients. For the intraoperative identification of an LLD, bilateral lesser trochanter position through the C-arm were compared. In addition, LLD was checked through the difference in length of both heels in the state of full extension of both lower extremities. Restoration of the LLD during surgery was considered important, but the operation was performed with the more important goal of maintaining implant stability.

### Radiographic measurements

Radiographic examination was performed within 1 week before surgery and at the last follow-up, which was at least > 6 weeks after surgery, when the LLD had stabilized. The reason for limiting the examination to > 6 weeks was to provide an accurate measurement of lower extremity length [8]. The interval between the questionnaire survey and the last X-ray follow-up date did not exceed 1 year. The imaging test was based on anteroposterior radiographic images of the pelvis, which the lower extremities were rotated internally by 15 degrees to adjust for size of the lesser trochanter and the obturator foramen on both sides.

To measure the LLD, the tip of the lesser trochanter was compared, based on the line connecting the lower ends of the teardrops on both sides [9]. This measurement was done by three reviewers, who were orthopedic surgeons not related to this study. Measurements were made twice for each case separated by an interval of 2 weeks. The average measured correlation coefficient for absolute agreement was calculated using a 2-way random-effects model, which had good intra-observer reliability (intraclass correlation coefficient [ICC] 0.996, 95% confidence interval [CI]: 0.993–0.997) and inter-observer reliability (ICC 0.875, 95% CI: 0.821–0.915). We stratified patients into two groups based on postoperative LLD of 5 mm. This division was predicated on the typical threshold at which patients perceive LLD following THA, as identified in previous studies [10].

### Clinical outcome survey

**Fig. 2:**
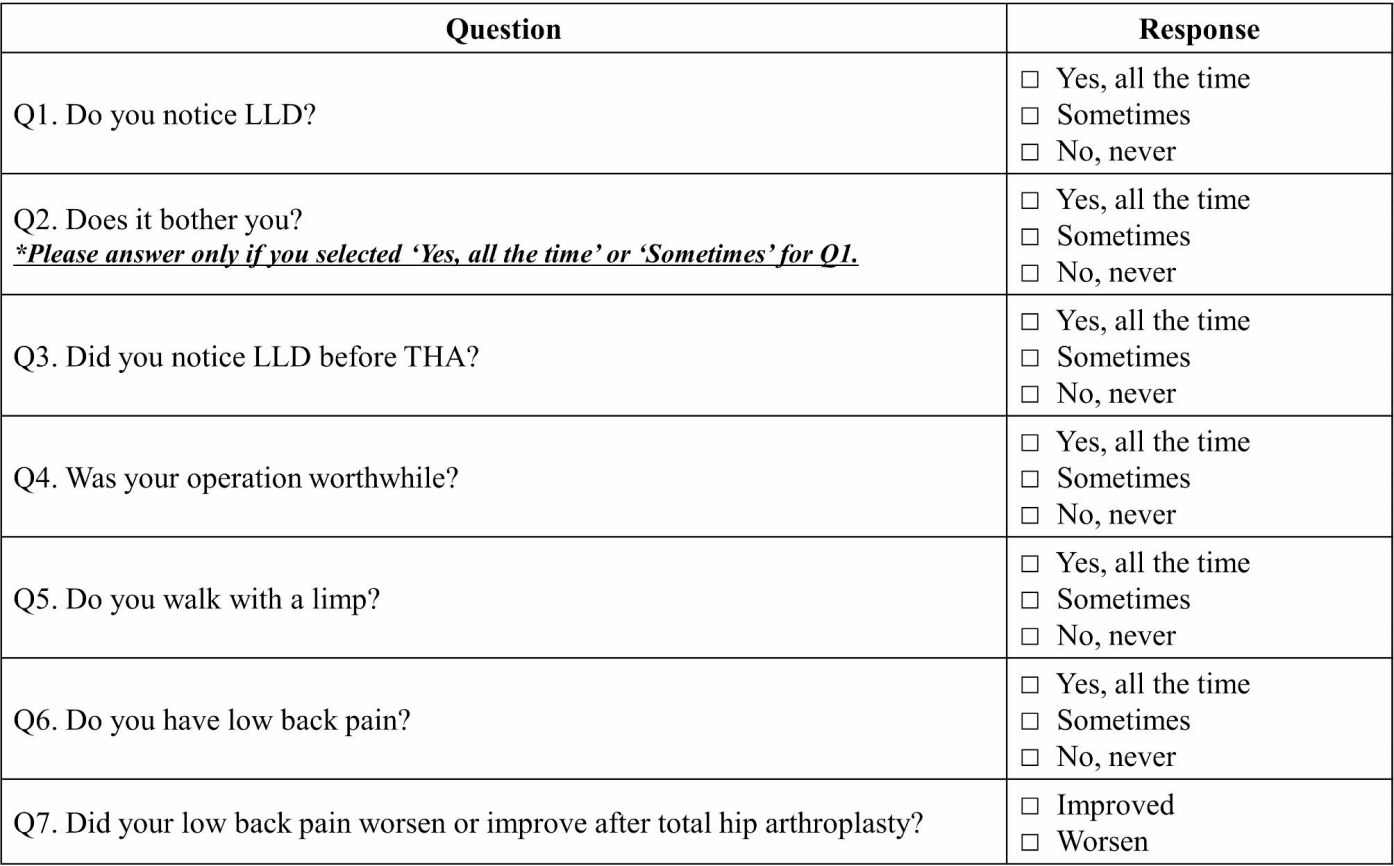
Questionnare designed to assess patient’s perception of LLD and their postoperative satisfaction; LLD = leg length discrepancy; THA = total hip arthroplasty

A questionnaire was used to ask patients whether they currently felt an LLD, and to ask patients about their satisfaction with THA. The survey items were determined by selecting relevant items from those previously used in studies on the LLD (Fig. 2) [11]. Two orthopedic surgeons not related to this study conducted the surveys.

As the primary outcome, discomfort according to the length of the lower extremities and satisfaction with the operation were confirmed. Secondary outcomes were patients’ subjective pain reduction and functional improvement, as determined by Harris Hip Score (HHS) and Western Ontario and McMaster Universities Osteoarthritis Index (WOMAC).

### Statistical analyses

For demographic data, categorical variables were evaluated using the Chi-square test or Fisher’s exact test, while continuous variables were assessed using the independent t-test. For each survey item, a Chi-squared test was performed to compare differences between groups. An independent t-test was used to evaluate differences between the HHS and WOMAC scores in each group. An alpha-level of P < 0.05 was considered statistically significant. IBM SPSS Statistics 28.0.1.1 (SPSS Inc., Chicago, IL, USA) was used for analysis.

## Results

41 patients were included in the group with LLD < 5 mm, and 21 patients were in the group with LLD of 5-10 mm. The mean follow-up period for 62 patients was 10.0 years, and the mean age at the time of surgery was 43.4 years. There were 59 males, accounting for 95.2% of patients. The mean body mass index was 24.6 kg/m^2^. Postoperative complications occurred in three patients in the group with LLD < 5 mm: wound dehiscence (n = 2), and discomfort due to LLD (n = 1). There was no significant difference in demographics including complication rate between two groups (Table 1). Among 62 patients who responded to the survey, there was no significant difference in the rate of subjective LLD after surgery between the group with an LLD of < 5 mm and the group with an LLD of 5-10 mm. There were significant differences in patient-reported satisfaction with the THA surgery (p = 0.035), and in limping gait during walking (p = 0.030). Additionally, back pain after arthroplasty was significantly improved in the patient group with an LLD of < 5 mm (p = 0.035) (Table 2). The average HHS after surgery in the group with an LLD of < 5 mm was 97.8, and in the other group, it was 89.3, showing a significant difference (p = 0.002). In addition, average WOMAC pain, WOMAC stiffness, and WOMAC physical function scores were 0.3, 0.1, and 1.8 in the group with an LLD of < 5 mm, and 2.2, 1.0, and 9.9 in the other group, respectively. Statistically significant differences were also confirmed in WOMAC pain (p = 0.003), stiffness (p = 0.007), and physical function scores (p = 0.009) (Table 3).

**Table 1:** Patient Demographics

	LLD < 5 mm (N = 41)	5 mm ≤ LLD < 10 mm (N = 21)	Total (N = 62)	P-value
**Age at surgery (year)** (Range)	41.6 (22 ~ 59)	46.9 (26 ~ 59)	43.4 (22 ~ 59)	0.064
**Sex**				0.263
Female	1	2	3	
Male	40	19	59	
**BMI (kg/m**^2^) (Range)	24.1 (16.4 ~ 38.6)	25.7 (20.8 ~ 42.4)	24.6 (16.4 ~ 42.4)	0.211
**Follow up duration (year)** (Range)	10.08 (1 ~ 18)	8.2 (1 ~ 18)	10.0 (1 ~ 18)	0.066
**Side**				0.788
Left	21	10	31	
Right	20	11	31	
**Approach**				0.110
MIS-2-inc	36	15	51	
Posterolateral	4	6	11	
**Lumbar spine fusion**				0.060
Complete	19	15	34	
Incomplete	22	6	28	
**Complications**				
Wound dehiscence	2	0	2	0.545
Revision due to LLD	1	0	1	1.000

LLD = leg length discrepancy; BMI = body mass index MIS-2-inc = Minimally invaisve 2 incision approach

**Table 2:** Group Differences in Survey Responses

Question	Response	LLD < 5 mm (N = 41)	5 mm ≤ LLD < 10 mm (N = 21)	P-Value
Q1. Do you notice LLD?				0.401
	Yes, all the time	7	3	
	Sometimes	9	8	
	No, never	25	10	
Q2. Does it bother you? *				0.718
	Yes, all the time	3	1	
	Sometimes	8	7	
	No, never	5	3	
Q3. Did you notice LLD before THA?				0.518
	Yes, all the time	15	5	
	Sometimes	2	2	
	No, never	24	14	
Q4. Was your operation worthwhile?				**0.035**
	Yes, all the time	41	18	
	Sometimes	0	0	
	No, never	0	3	
Q5. Do you walk with a limp?				**0.030**
	Yes, all the time	0	3	
	Sometimes	13	8	
	No, never	28	10	
Q6. Do you have low back pain?				0.819
	Yes, all the time	7	4	
	Sometimes	15	6	
	No, never	19	11	
Q7. Did your low back pain worsen or improve after THA?				**0.035**
	Improved	41	18	
	Worsen	0	3	

*This question was only surveyed to those who answered ‘Yes,all the time’ or ‘Sometimes’ for Q1; LLD = leg length discrepancy; THA = total hip arthroplasty

**Table 3:** Group differences in post operative clinical score

	LLD < 5 mm (N = 41)	5 mm ≤ LLD < 10 mm (N = 21)	P-Value
**Mean HHS** (Range)	97.8 (88 ~ 100)	89.3 (42 ~ 100)	0.002
**Mean WOMAC**			
Pain (Range)	0.3 (0 ~ 4)	2.2 (0 ~ 15)	0.003
Stiffness (Range)	0.1 (0 ~ 2)	1.0 (0 ~ 4)	0.007
Physical function (Range)	1.8 (0 ~ 17)	9.9 (0 ~ 51)	0.009

LLD = leg length discrepancy; HHS = Harris Hip Score; WOMAC = Western Ontario and McMaster Universities Osteoarthritis Index

## Discussion

When patients were divided according to an LLD of 5 mm after THA, statistically significant differences were evident in the incidence of limping gait and patient-reported satisfaction with the operation. In addition, patients with an LLD of < 5 mm after THA reported a significant improvement in back pain compared with the group with an LLD of 5-10 mm. Röder et al. reported that LLD > 10 mm after THA significantly affected postoperative limping gait, back pain, and surgical satisfaction [12]. Sykes et al. also reported that patients can recognize LLD when it is more than 5 mm after THA [10].

**Fig. 3:**
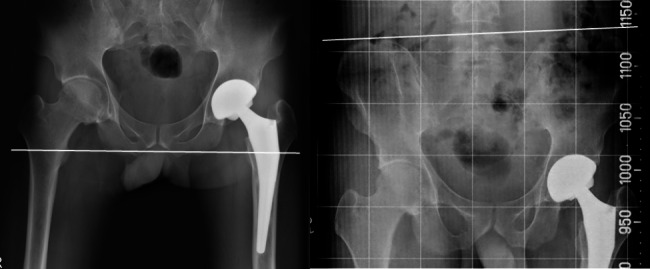
Patient with pelvic obliquity

In patients with remaining spine facet joint movement, trunk imbalance can be resolved with a compensatory action due to the movement of each spine facet joint. However, since patients with ankylosing spondylitis have partial or complete fusion of the spine, trunk balance is not compensated. Therefore, an LLD of ≥ 5 mm may cause patient dissatisfaction with the operation. An LLD of ≥ 5 mm can also cause a functional limping gait. In addition, the statistically significant improvement in lower back pain in patients whose LLD was corrected to < 5 mm suggests that the imbalance caused by LLD is related to low back pain in patients with ankylosing spondylitis and a fused spine. Even when there was an LLD of ≥ 5 mm on radiographic examination, many patients answered in the survey that they did not feel the difference. However, eight patients reported perceived LLDs, even though the radiographic examination revealed an LLD of < 5 mm. Notably, these eight patients presented with pelvic obliquity. Fujita et al. reported that patients with pelvic obliquity who had their radiographic LLD accurately corrected often experienced increased discomfort due to the LLD [13]. Similarly, our findings also suggest that precise correction of LLD can lead to heightened inconvenience in such patients. (Fig. 3). This suggests that surgery that considers only the LLD may cause more discomfort in patients with ankylosing spondylitis with pelvic obliquity rather than without pelvic obliquity. For these patients, the goal is not to correct the LLD accurately after surgery, but to make the LLD to maintain the body balance after surgery is thought to be helpful in improving the postoperative result.

Although this study has limitations as a retrospective study, it was impossible to conduct a prospective study due to the patient group and research topic. Although there are many cases of hip arthritis in patients with ankylosing spondylitis, the number of cases requiring surgical treatment is limited; thus, the number of patients in each group was small, so it might affect the generalizability of our findings. For patients’ evaluation, only a simple radiographic examination was performed to measure patients’ postoperative lower extremity length. In patients with ankylosing spondylitis, where the spine and sacroiliac joints are fused and the vertebral joints have less compensatory function, a more accurate difference could be determined if balance was measured through a whole-body spinal radiograph or gait test that considered whole-body balance, and not just the LLD. All patients reported that they did not wear insoles, even though insoles can improve a limping gait and discomfort; however, recommendations or guidance from doctors about insole use may have been insufficient. Additionally, the absence of multivariate analyses means potential confounding variables might not have been adequately addressed. Future studies should consider these limitations to enhance the robustness and applicability of their findings.

## Conclusions

Previous studies have emphasized the importance of the LLD after THA, but there has been no study on the extent of LLD after THA in patients with ankylosing spondylitis. When performing THA for patients with ankylosing spondylitis, it is necessary to perform surgery to a stricter LLD standard of < 5 mm, compared to patients with general arthritis, as spinal compensation is insufficient in patients with ankylosing spondylitis, who may feel severe discomfort and dissatisfaction with THA.

## Data Availability

The datasets generated and/or analysed during the current study are available from the corresponding authors upon reasonable request.

## References

[1] Raychaudhuri SP, Deodhar A (2014). The classification and diagnostic criteria of ankylosing spondylitis. J Autoimmun.

[2] Vander Cruyssen B, Muñoz-Gomariz E, Font P, Mulero J, de Vlam K, Boonen A, Vazquez-Mellado J, Flores D, Vastesaeger N, Collantes E (2010). Hip involvement in ankylosing spondylitis: epidemiology and risk factors associated with hip replacement surgery. Rheumatology (Oxford).

[3] Blizzard DJ, Penrose CT, Sheets CZ, Seyler TM, Bolognesi MP, Brown CR (2017). Ankylosing Spondylitis increases Perioperative and Postoperative Complications after total hip arthroplasty. J Arthroplasty.

[4] Wylde V, Whitehouse SL, Taylor AH, Pattison GT, Bannister GC, Blom AW (2009). Prevalence and functional impact of patient-perceived leg length discrepancy after hip replacement. Int Orthop.

[5] Renkawitz T, Weber T, Dullien S, Woerner M, Dendorfer S, Grifka J, Weber M (2016). Leg length and offset differences above 5mm after total hip arthroplasty are associated with altered gait kinematics. Gait Posture.

[6] Yoon TR, Bae BH, Choi MS (2006). A modified two-incision minimally invasive total hip arthroplasty: technique and short-term results. Hip Int.

[7] Park KS, Yoon TR, Hwang SY, Lee KB (2012). Modified minimally invasive two-incision total hip arthroplasty using large diameter femoral head. Indian J Orthop.

[8] Smolle MA, Fischerauer SF, Maier M, Reinbacher P, Friesenbichler J, Ruckenstuhl P, Grandesso M, Leithner A, Maurer-Ertl W (2021). Leg length measures appear inaccurate in the early phase following total hip arthroplasty. Sci Rep.

[9] Sayed-Noor AS, Hugo A, Sjödén GO, Wretenberg P (2009). Leg length discrepancy in total hip arthroplasty: comparison of two methods of measurement. Int Orthop.

[10] Sykes A, Hill J, Orr J, Humphreys P, Rooney A, Morrow E, Beverland D (2015). Patients’ perception of leg length discrepancy post total hip arthroplasty. Hip Int.

[11] Oommen AT, Hariharan TD, Chandy VJ, Poonnoose PM, Kuruvilla AAS, Timothy RS (2021). Total hip arthroplasty in fused hips with spine stiffness in ankylosing spondylitis. World J Orthop.

[12] Röder C, Vogel R, Burri L, Dietrich D, Staub LP (2012). Total hip arthroplasty: leg length inequality impairs functional outcomes and patient satisfaction. BMC Musculoskelet Disord.

[13] Fujita K, Kabata T, Kajino Y, Tsuchiya H (2020). Optimizing leg length correction in total hip arthroplasty. Int Orthop.

